# Audio-Visual Training in Older Adults: 2-Interval-Forced Choice Task Improves Performance

**DOI:** 10.3389/fnins.2020.569212

**Published:** 2020-11-12

**Authors:** Jessica M. O’Brien, Jason S. Chan, Annalisa Setti

**Affiliations:** School of Applied Psychology, University College Cork, Cork, Ireland

**Keywords:** perceptual learning, multisensory, audio-visual, sound-induced flash illusion, training, aging

## Abstract

A growing interest in ameliorating multisensory perception deficits in older adults arises from recent evidence showing that impaired multisensory processing, particularly in the temporal domain, may be associated with cognitive and functional impairments. Perceptual training has proved successful in improving multisensory temporal processing in young adults, but few studies have investigated this training approach in older adults. In the present study we used a simultaneity (or synchronicity) judgement task with feedback, to train the audio-visual abilities of community-dwelling, cognitively healthy older adults. We recruited 23 older adults (*M* = 74.17, *SD* = 6.23) and a group of 20 young adults (*M* = 24.20, *SD* = 4.23) who served as a comparison. Participants were tested before and after perceptual training using a 2-Interval Forced Choice Task (2-IFC); and the Sound-Induced Flash Illusion (SIFI). After 3 days of training, participants improved on the 2-IFC task, with a significant narrowing of the temporal window of integration (TWI) found for both groups. Generalization of training effects was not found, with no post-training differences in perceptual sensitivity to the SIFI for either group. These findings provide evidence perceptual narrowing can be achieved in older as well as younger adults after 3 days of perceptual training. These results provide useful information for future studies attempting to improve audio-visual temporal discrimination abilities in older people.

## Introduction

The appropriate integration of inputs coming from the different senses is a crucial ability to support an efficient interaction with the environment (Alais et al., 2010; Freiherr et al., 2013; Murray et al., 2016; de Dieuleveult et al., 2017). One of the ways in which the brain determines the appropriateness of this integration is based on the temporal features of the incoming stimuli; if the inputs from different senses fall within the temporal window of integration (TWI), they are combined into a unitary percept; otherwise they are processed as separate events (Vroomen and Keetels, 2010). In turn, the size of the TWI is associated with our sensitivity in discriminating the order of temporally asynchronous stimuli (Stevenson et al., 2012), which can be determined with tasks such as temporal order judgments (TOJ) or synchronicity judgments (Chan et al., 2014b; Setti et al., 2014; Bedard and Barnett-Cowan, 2016; Scurry et al., 2019; Theves et al., 2020).

Older adults show larger temporal order thresholds (i.e., the minimum temporal interval leading to accurate discrimination) than younger adults (Bedard and Barnett-Cowan, 2016), enhanced susceptibility to audio-visual illusions (Setti et al., 2011; McGovern et al., 2014), increased difficulty judging audio-visual synchrony (Chan et al., 2014a), and reduced audio-visual recalibration compared to young adults (Chan et al., 2014b). They display an enlarged TWI (Colonius and Diederich, 2004; Diederich et al., 2008), and enhanced benefits in perceiving congruent multisensory stimuli, with cognitive (Laurienti et al., 2006), and functional benefits (Mahoney and Verghese, 2018). However, an enlarged TWI may be disadvantageous when inputs from different senses are incongruent, as shown utilizing the Sound-Induced Flash Illusion (SIFI; Setti et al., 2011; Chan et al., 2015). A wider TWI in older adults could be related to their decreased unisensory abilities, as, in line with the principle of Inverse Effectiveness, greater gain is obtained by combining weaker unisensory stimuli (Holmes, 2007). According to the TWI model (Colonius and Diederich, 2011) integration occurs very rapidly. The start of peripheral processing of sensory inputs (regardless of modality) triggers the opening of the TWI; when the SOA between the unisensory stimuli is sufficiently short to allow for completion of the processing of both stimuli within the window, then the inputs are integrated. Multisensory integration is thought to occur based on the likelihood reliability of inputs from the different modalities for the task at hand (Maximum Likelihood Estimation and Information Reliability (Schwartz et al., 1998). Therefore, reduced unisensory ability would change the likelihood of integration and enhance reliance on prior knowledge, or perceptual priors (Ernst and Banks, 2002; Chan et al., 2017), which is associated with a wider TWI in older adults.

Considering the potential benefits of efficient multisensory integration (Murray et al., 2016), refining the TWI by enhancing the temporal discrimination abilities of individuals presents a potential avenue for supporting or improving cognitive and functional abilities in older adults with cognitive and functional deficits (Setti et al., 2011; Chan et al., 2015; Mahoney and Verghese, 2018; Murray et al., 2018; Hernández et al., 2019).

In healthy young adults, cross-sensory temporal discrimination abilities have been successfully improved through perceptual training (Powers et al., 2009, 2016) by exposing participants to pairs of stimuli separated by different temporal intervals and asking them to determine either: which one came first, the visual or the auditory, or whether they were synchronous and providing informative feedback. Specifically, such training paradigms included asking participants to determine the temporal order of stimuli (Temporal Order Judgment, TOJ), just asking whether stimuli were synchronous (2-AFC), or judging which pair of stimuli of two sets were presented simultaneously (2-interval forced choice, 2-IFC; see Stevenson and Wallace, 2013). Powers et al. (2009) utilized two training tasks, a two-alternative forced choice task (2-AFC) and a two-interval forced choice (2-IFC) task. In the first paradigm, participants are asked to decide whether two stimuli (one visual and one auditory) appeared simultaneously or not (2-AFC). In the second paradigm, participants are exposed to two sets of audio-visual stimuli, and they are asked to decide in which pair, the first or the second, the stimuli were simultaneous (2-IFC). The 2-IFC paradigm has also been used in two variants: with one pair always portraying physical simultaneity (Stevenson and Wallace, 2013) or where both pairs are not simultaneous and the task was to indicate which pair was closer to simultaneity (Yarrow et al., 2016). In Powers et al. (2009) the first type of 2-IFC was utilized. During training, feedback was provided on whether the participant answered correctly. Young participants were trained for 5 days, and with both paradigms they had a marked narrowing of the TWI. In a follow up study (Powers et al., 2012), participants showed a significant narrowing of the TWI after only 1 day of 2-IFC training. This behavioral improvement was coupled by decreases in BOLD activity post-training in the posterior Superior Temporal Sulcus (pSTS) and in primary visual and auditory areas of the cortex (Powers et al., 2012). This decrease in BOLD activity can be associated with an increase in beta-band activity, as they have been shown to have a negative relationship (Conner et al., 2011; Hanslmayr et al., 2011). Furthermore, using dynamic causal modeling, Powers et al. (2012) found effective connectivity between sensory areas and pSTS changed from a mainly feed-forward model (pre-training) to a more evenly distributed effective connectivity between brain areas, post-training. It has been suggested in some neural models (e.g., predictive coding) that increased beta-band activity represents increased template information (Mumford, 1992; Arnal and Giraud, 2012; Bastos et al., 2012; Bastos, 2013), which would be in line with these results (Powers et al., 2012). More recently, Theves et al. (2020) used magnetoencephalography (MEG) to investigate the neural oscillations associated with a reduced TWI. Using the same paradigm as Powers et al. (2012), they found increased beta-band activity (12–25 Hz) post-training compared to pre-training. Therefore, the increase in beta-band activity after training can be due to improved perceptual models associated with the TWI (Theves et al., 2020).

In relation to older adults, the work of Theves et al. (2020) is promising, as increased perception in the SIFI was associated with an overall increase in beta-band activity in younger adults (Theves et al., 2020). One would expect that perceptual training could potentially modify effective connectivity in *older* adults as well (with a related modulation of beta-band activity) although to test this hypothesis is beyond the scope of the present study. Older adults have also shown to be susceptible to audio-visual training. Using a different paradigm (i.e., an audio-visual temporal order judgment utilizing a staircase procedure), Setti et al. (2014) found narrowing of the TWI in healthy older participants was possible, although not all individuals actually showed the beneficial effects of training. In the same study, older participants who did show a narrowing of the TWI also showed decreased susceptibility to the SIFI, indicating potential benefits to appropriately discriminate or integrate multisensory stimuli in a different task. However, with the 2-IFC task by Powers et al. (2016), a sample of young adults had only limited benefits in terms of susceptibility to the SIFI, i.e., benefits were related to when there was the same number of beeps and flashes, not when they were incongruent.

Considering older adults’ exhibit inefficient multisensory processing in the form of an enlarged TWI (Laurienti et al., 2006) and emerging evidence that this perceptual function is critical for balance control and functional mobility (Mahoney et al., 2019; Zhang et al., 2020), identifying methods to promote efficient multisensory processing in older adults is paramount. In the present study, we investigate whether perceptual training which has proved beneficial for younger adults’ multisensory processing capabilities (i.e., Simultaneity Judgment training) is effective for older adults. We adopted the 2-IFC paradigm with a sample of community-dwelling older adults to test, behaviorally, whether a reduction of the TWI can be obtained in older adults and if it can be associated with a reduction in susceptibility to the SIFI. While there is some evidence that lifestyle factors, such as physical exercise, may modulate SIFI perception in older adults (O’Brien et al., 2017); lab-based perceptual training, at present, appears to be a partially successful way to narrow the TWI and modulate susceptibility to the SIFI (Setti et al., 2014), which, in turn, is associated with cognitive performance and mobility. The literature on multisensory perceptual training in older adults is still limited, deserving further investigation.

We hypothesized that if the 2-IFC can be used successfully with older adults we should find training benefits after 3 days of targeted perceptual training. If training is successful, we expect improved performance on the 2-IFC task and reduced TWI in participants’ who train. In order to assess for the generalizability of the training, we also tested participants on the SIFI, in line with Setti et al. (2014), and hypothesized that following successful training, participants should be less susceptible to the illusion. In addition, to ensure that the training protocol could replicate the successful results of Powers et al. (2009) with younger adults, we tested a group of young adults with the same methodology utilized for older adults, expecting young adults to improve in their performance on the 2-IFC task following training. We also hypothesize that training should generalize to improved sensitivity on the SIFI task in line with studies by Setti et al. (2014) and Powers et al. (2016) finding benefits for SIFI performance following perceptual training.

## Materials and Methods

### Participants

Two groups of participants were recruited: an older adult group and young adult group. Twenty-three older adults (10 female) aged between 60 and 85 (*M* = 74.17, *SD* = 6.23) participated. These participants were living independently in the community at the time of the study. The young adult group comprised 20 adults aged between 18 and 35 (*M* = 24.20, *SD* = 4.23). All participants reported normal or corrected-to-normal vision and hearing. Older participants were recruited through local active retired groups, community groups and community Falls groups. An analysis of the older group found no significant difference between those considered fallers (*n* = 11) and non-fallers (*n* = 12) on mean scores on any of the SIFI unisensory control conditions or dependent variables i.e., performance on 2-IFC task or SIFI task; *p* > 0.8). Young adult participants were recruited through university mailing lists and snowball recruiting. Data collection for the older adult group yielded a total of 28 participants, 5 were excluded from analyses procedures; 4 did not complete the full 3 day experimental protocol and 1 participant was identified as an outlier on control conditions of the SIFI (indicating poor sensory acuity or misunderstanding of the task instructions). See Table 1 for descriptive characteristics for each group.

**TABLE 1:** Descriptive characteristics per training group (mean values with standard deviations in parentheses).

	Older	Younger	*p*-value
*N*	23	20	
Age	74.17 (6.23)	24.20 (4.23)	–
Gender	M: 13 (56.52%), F: 10 (43.48%)	M: 9 (45%), F: 11 (55%)	0.45

No participants reported any psychiatric or neurological conditions that would preclude them from the study. The older adults (*n* = 23) and young adults (*n* = 20) did not significantly differ in their gender identification (*Pearson chi square* = 0.57, *p* = 0.45).

We conducted research on a separate cohort of participants (*n* = 20) investigating whether training that focused on the asynchrony of stimuli, instead of their simultaneity would be more beneficial (i.e., more similar to a TOJ’s task demands, as TOJ was utilized by Setti et al., 2014). Due to the global Covid-19 pandemic, data collection for a young group on this alternative training protocol was not possible, please see Supplementary Material for data on this older asynchronous group.

Ethical approval was granted by the Ethics Committee at the School of Applied Psychology, University College Cork. All guidelines set out by the Declaration of Helsinki were adhered to.

### Demographic Data

Data on participants’ age, gender and regular physical activity levels (IPAQ; Booth, 2000) were collected via self-report questionnaires. Additional questionnaires for the older adult group collected information on falls-risk and global cognitive health (SMMSE). The questionnaire on falls-risk asked participants to rate their steadiness while seated, standing, in motion etc. This study is part of a wider study on falling, the data on falls are not utilized for the purpose of this study.

### Baseline Measures

#### Sound-Induced Flash Illusion (SIFI)

The Sound-induced Flash Illusion (SIFI; Shams et al., 2002) was used to assess participants’ multisensory integration efficiency. The SIFI consists of the illusory perception of two flashes (a white dot subtending 2° of visual angle, presented for 16 ms at 8° of visual angle below the fixation cross on a black screen background) when one flash is presented simultaneously with two beep sounds (3,500 Hz, duration 16 ms, 20 μs rise-and-decay, 68 dBA sound pressure level). The SIFI task was administered using a Dell desktop computer (24-inch screen). The software suite Presentation (version 18; Neurobehavioral Systems) was used to program and administer the SIFI. Volume and brightness were kept constant for all participants. All participants reported no difficultly in seeing or hearing the stimuli. Participants were seated for the task, approximately 50 cm from the computer screen. Closed-back, circumaural headphones (AKG K271) delivered the auditory stimuli. For the task, participants were asked to report the number of flashes seen on the computer screen. Responses were made using the keyboard numbers (0, 1, 2, etc.).

Control unisensory trials (single flash or double flash trials and single beep or double beep trials) were also presented. The trials of interest were the illusory trials, where one flash was presented with two beeps. The stimulus onset asynchrony (SOA) between the stimuli (i.e., between first the flash/beep pair and second beep) varied according to the trial. The SOAs were 70, 110, 150, and 230 ms. The visual-only and multisensory trials were presented 6 times per SOA condition (total of 84 trials). The auditory only trials were presented in a separate block, twice per condition, 1 beep and 2 beeps (at each SOA) for a total of 10 trials. The measurement of interest was the mean proportion of correct responses to illusory trials. Participants fixated on a white central fixation cross (1 × 1 cm) presented on a black background. The fixation cross remained constant throughout the task, disappearing only for the presentation of trials.

#### Pre- and Post-training 2-IFC Task

Participants’ ability to discriminate perceptual synchrony was assessed using a 2-IFC task as in Powers et al. (2009). The same computer and apparatus were used to administer this task as described for the SIFI task. During the task, participants were asked to judge which pair of stimuli are synchronous or occurred simultaneously. For each trial in this task, participants were presented with two pairs of stimuli; each pair had a white annulus and an auditory beep. In each trial, one pair was always presented synchronously while the other was asynchronous. There was a 1,000 ms gap in between each interval. The asynchronous pairs varied by SOA (50, 100, 150, 200, 250, 300 ms). The choice of SOAs for the training reflects those utilized in successful training with younger adults, and it is in line with Setti et al. (2014). In their study, also conducted with older adults, the audio-visual temporal order judgment (TOJ) staircase converged at an SOA of approximately 250 ms before training. After 5 days of audio visual TOJ training, the staircase converged at an SOA of approximately 150 ms (depending on the group), therefore it was plausible to hypothesize that older adults would be able to perform the task, at least at the longer SOAs (200, 250, 300 ms). Participants were instructed to select the interval pair in which the stimuli were presented synchronously (i.e., simultaneously). Participants responded verbally (first or second pair) and the experimenter recorded the response using the computer keyboard (pressing either the “1” or “2” button). A response was required for each trial. Participants were instructed to give their best guess if they were unsure of the answer. Participants did not receive feedback after each response.

For the visual stimulus, a white annulus surrounding the fixation cross (8.8 degrees of visual angle at 50 cm distance) was utilized and presented for 16 ms against a black background. The auditory beep was a 1,800 Hz tone with duration 16 ms (Powers et al., 2012). The stimulus was presented to both ears via circumaural headphones (AKG K271; audibility was subjectively checked with participants). Visual and auditory stimuli were presented at SOAs ranging from 50 to 300 ms at 50 ms intervals, with visual-lead only. Each condition was repeated 24 times. The set of stimuli was kept constant for all participants (see Powers et al., 2009) for additional methodological details of this task.

### Perceptual Training Protocol

**FIGURE 1:**
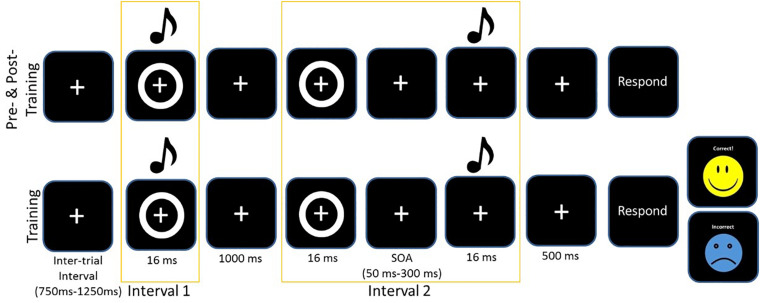
Example of one trial of the 2-IFC task for the assessment pre/post and the training. Asynchronous trials always presented the visual stimulus first. The training protocol included feedback.

The training protocol consisted of completing the same 2-IFC task but with informative feedback after each trial. After each response, feedback was given on screen with the word “correct” and a yellow smiley face appearing on screen or if the participant selected the wrong pair, the word “incorrect” would appear on screen with a blue sad face (see Figure 1). Again, participants were instructed to select the stimulus pair which was synchronous (i.e., the pair in which the circle and beep occurred simultaneously). All participants had a 5 min break during the training to combat fatigue. As with the perceptual task and the SIFI task, participants responded verbally, and the experimenter entered responses into the computer. We chose to train participants for 3 days, as the 2-IFC had been a successful paradigm with younger adults for both 5 days and 1 day, and pilot work for Setti et al. (2014) suggested that 3 days was the minimum to register positive change in older adults. Figure 1 illustrates the training and test protocols. For further details on the Training task, see (Powers et al., 2009).

### Procedure

Participants completed three successive testing sessions, Days 1, 2, and 3. On Day 1, participants completed the demographics questionnaires and the two pre-training measures (SIFI and 2-IFC Task) followed by the first session of training. On Day 2, participants completed the second session of training. On Day 3, participants completed the third session of training followed by the two post-training measures. Participants were tested individually in a quiet room. For all tasks, participants were seated in front of the computer screen with the experimenter sitting beside them. Participants who normally wore glasses or contact lenses wore them for the experiment.

The order of the pre-training test was consistent across participants. Participants always completed the SIFI first, followed by the 2-IFC task and the training protocol. At post-training, participants completed the final training session, followed by the 2-IFC post-training task and the SIFI, in that order.

### Analysis

Analysis of Variance was utilized to compare the groups when assumptions were met. Greenhouse-Geisser corrections were applied where Mauchly’s test of sphericity was breached. Non-parametric statistical analyses were utilized to account for the skewed distribution of the TWI and of the SIFI illusory data. For each non-parametric ANOVA, care was taken to define the appropriate permutations for a factorial design (see Anderson and Braak, 2003 for methods; Suckling and Bullmore, 2004). To avoid confounds due to the within-participant factor when estimating main effects, observations for both levels of within-participant factors were kept together for each participant. Only whole participants were allowed to be exchanged during the permutation procedure. No exact permutation tests, based on the F-statistic, exist for the interaction effect, since restricting permutation of the observations such that neither group nor condition main effect affects the corresponding F-ratio, would leave no possible permutations of the data. An approximate test can be constructed by restricting permutations of condition levels to occur within participant and subsequently permuting whole participants across groups (Anderson and Braak, 2003; Suckling and Bullmore, 2004; Theves et al., 2020).

In the SIFI task, the dependent variable was *d’* scores, which reflects perceptual sensitivity and performance on the SIFI task (Chan et al., 2014b; McGovern et al., 2014; Setti et al., 2014). *d’* captures a participant’s ability to correctly perceive 2 flashes when they are real (i.e., correctly detecting 2 flashes in 2flash/2beeps conditions) relative to their susceptibility to the illusion (i.e., incorrectly perceiving 2 flashes in 1flash/2beeps conditions). Sensitivity was calculated using the following formula: *d’* [z(hits) – z(false alarms)]; where hits were the proportion of correct responses on 2flash/2beeps conditions and false alarms were the proportion of incorrect responses to 1flash/2beeps conditions. *z* refers to the inverse cumulative norm (Green and Swets, 1966).

## Results

### 2-IFC Task

A three-way mixed ANOVA was performed on the mean proportion of correct scores per SOA (e.g., mean value of 0.2 represents 20% correct scores on the task). Group (younger, older) was the between-participants factor and Time (pre-training vs. post-training) and SOA were the within-participants factors. There was a significant main effect of Group [*F*(1, 41) = 41.21, *p* < 0.001, $\eta_{\text{p}}^{2}$ = 0.5], Time [*F*(1, 41) = 12.31, *p* < 0.001, $\eta_{\text{p}}^{2}$ = 0.23] and SOA [*F*(3.25, 133.14) = 64.01, *p* < 0.001, $\eta_{\text{p}}^{2}$ = 0.61]. The main effect of Group was driven by the young group having higher mean scores (proportion of correct answers) than the older group. The main effect of Time was due to both groups having higher mean proportion correct at post-training compared to pre-training (younger: pre-training *M* = 0.73, *SD* = 0.09; post-training *M* = 0.78, *SD* = 0.68; older: pre-training *M* = 0.56, *SD* = 0.09; post-training *M* = 0.62, *SD* = 0.12). The main effect of SOA was expected, with higher mean proportion correct at longer SOAs and lower mean scores at shorter SOAs. The interaction between SOA and Group was significant [*F*(3.25, 133.14) = 12.59, *p* < 0.001, $\eta_{\text{p}}^{2}$ = 0.24] with the young group having significantly higher mean scores for each SOA compared to the older group (*p* < 0.05). There was no interaction between Group and Time [*F*(1, 41) = 0.14, *p* = 0.72, $\eta_{\text{p}}^{2}$ = 0.003]. There were no other significant interactions.

**FIGURE 2:**
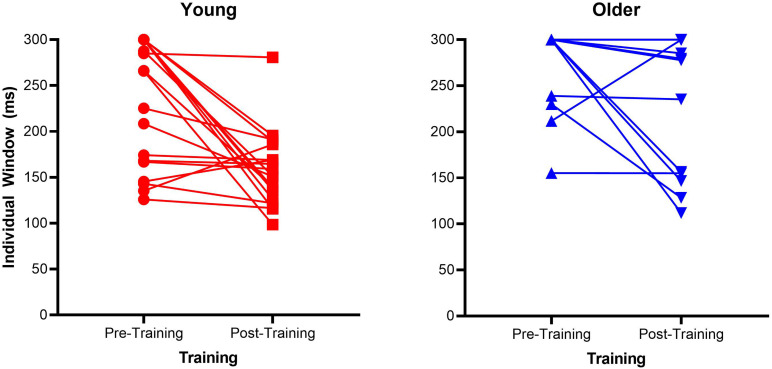
Scatterplots of individual Temporal Window of Integration (TWI) pre- and post-training for the older adult and young adult groups. The dotted lines represent the fitted lines for individual data.

Mean proportion of correct responses for each SOA is a very crude measure of the TWI, therefore we calculated the size of the individual windows, a 3rd order polynomial probability curve was fitted across the SOAs for each participant. The size of each participant’s TWI was defined as the SOA at an accuracy level halfway between the individuals’ lowest accuracy point and 100% (as in Theves et al., 2020). Supplementary Figure S1 depicts the calculation of the TWI for one participant. See Figure 2 for a scatterplot of each individual TWI. In order to analyze the TWI, a non-parametric 2 × 2 mixed ANOVA using 10% trimmed means was conducted. Group (older vs. younger) and Time (pre- vs. post-training) were the respective between-participants and within-participants factors. There was a main effect of Group [*F*(1, 26.42) = 34.32, *p* < 0.001, $\eta_{\text{p}}^{2}$ = 0.46] and a main effect of Time [*F*(1, 26.46) = 10.95, *p* = 0.003, $\eta_{\text{p}}^{2}$ = 0.21]. Additionally, there was a significant interaction between these two factors [*F*(1, 26.46) = 4.72, *p* = 0.038, $\eta_{\text{p}}^{2}$ = 0.1]. To follow-up the significant interaction effect, non-parametric Wilcoxon Signed-Rank tests were conducted on each group separately with Time as the within-participants factor (pre-, post-training). This test confirmed the older groups’ TWI significantly reduced (*Z* = 6, *p* = 0.03) from pre (*M* = 284.165, *SD* = 37.92) to post test (*M* = 259.84, *SD* = 66.8), as did the young group (*Z* = 24.85, *p* = 0.001), with a significantly reduced TWI at post-training (*M* = 165.05, *SD* = 51.01) compared to pre-training (*M* = 232.86, *SD* = 67.93). The daily training is presented in Supplementary Figure S2.

### Sound-Induced Flash Illusion: Pre- and Post-training

The proportion of correct responses to the SIFI task are reported in Table 2. Prior to assessing participants’ susceptibility to the SIFI, it was first necessary to determine their ability to perceive the auditory and visual stimuli used during the task. To this end, we analyzed participants’ ability to perceive unisensory conditions (1 flash, 2 flashes, 1 beep, 2-beeps trials) and non-illusory multisensory conditions (2 flash and 2 beeps trials) at baseline (pre-training) using Mann-Whitney *U*-tests. There were no group differences in perceiving one flash (*U* = 240, *p* = 0.35), one beep (*U* = 240, *p* = 0.35), nor one-flash/one-beep, whereby all participants scored 100% correctly. There were no significant differences between the two groups on the multisensory control conditions (2 flashes/2 beeps) at each SOA.

**TABLE 2:** Sound-induced flash illusion: mean and standard deviation (in parentheses) for the proportion of correct in the illusion conditions and *d’* score.

	Older	Younger
SOA	*M*	*M*
**Pre**		
70	0.42 (0.36)	0.48 (0.29)
110	0.33 (0.4)	0.5 (0.37)
150	0.27 (0.33)	0.65 (0.36)
230	0.27 (0.32)	0.87 (0.24)
**Post**		
70	0.39 (0.24)	0.44 (0.26)
110	0.19 (0.27)	0.51 (0.29)
150	0.23 (0.28)	0.75 (0.33)
230	0.3 (0.31)	0.93 (0.23)
***d***’ **Pre**		
70	0.8 (1.05)	1.5 (0.89)
110	1.05 (1.52)	1.93 (1.47)
150	1.19 (1.49)	2.78 (1.71)
230	1.29 (1.65)	3.75 (1.25)
**Post**		
70	0.54 (1.29)	1.22 (0.88)
110	0.27 (1.64)	2.15 (1.31)
150	0.67 (1.4)	3.44 (1.44)
230	1.06 (1.72)	4.01 (1.47)

There was a group difference in both unisensory control conditions (2-flashes and 2-beeps conditions). Groups significantly differed on performance on 2-flashes (*U* = 361.5, *p* = 0.001), with the older group registering significantly fewer correct responses (average accuracy of 78%) than their younger counterparts (over 90% accuracy). Of note, both groups had significantly poorer accuracy at SOA 70 ms compared to longer SOAs (mean accuracy, older = 53%; younger = 76%). For the 2-beeps condition, there was a significant difference in mean scores (*U* = 294.5, *p* = 0.047), however this difference should be interpreted with caution as 9 participants were excluded due to missing data for SOA 110 ms due to a technical error. Group differences were due to the older group registering lower accuracy on the 2-beeps condition (average accuracy of 92.75% at pre-training and 88.5% at post-training) compared to the younger group (over 98% accuracy at pre- and post-training). The group differences in discriminating unisensory conditions was noted and is acknowledged in the discussion. See Supplementary Table S1 for group means on each unisensory condition.

**FIGURE 3:**
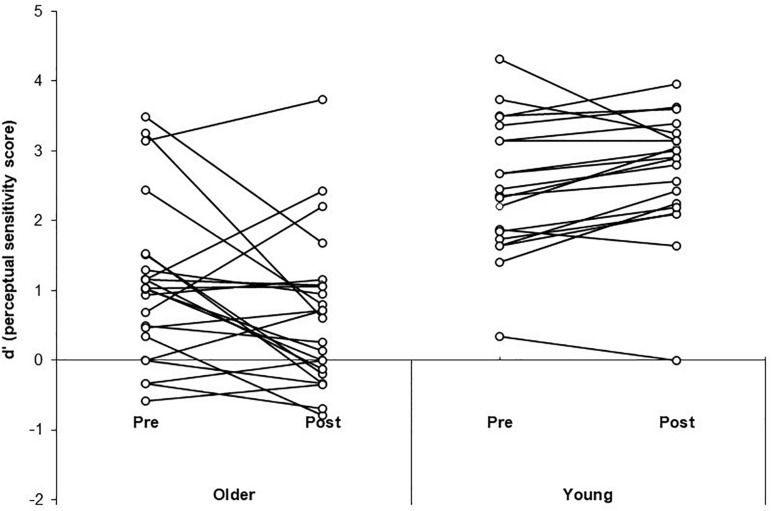
Scatterplots of individual perceptual sensitivity scores (*d*’) pre- and post-training for the older adult and young adult groups.

A three-way Mixed ANOVA was performed on the mean *d’* scores for the illusion condition, with Time (pre-training, post-training) and SOA (70, 110, 150, 230 ms) as the within-participants factors and Group (older, younger) as the between-participants factor. There was no significant main effect of Time [*F*(1, 41) = 0.83, *p* = 0.37, $\eta_{\text{p}}^{2}$ = 0.02]. There was a significant main effect of Group [*F*(1, 41) = 37.35, *p* < 0.001, $\eta_{\text{p}}^{2}$ = 0.48] with simple contrasts revealing young adults performed better (i.e., had higher perceptual sensitivity scores) than the older group [*contrast estimate* = 0.66, *p* = 0.02]. There was a significant main effect of SOA [*F*(3, 123) = 24.75, *p* < 0.001, $\eta_{\text{p}}^{2}$ = 0.38]. Simple contrasts revealed participants performed significantly better at the longest SOA (230 ms) compared to shorter SOAs 70 and 150 ms (*p* < 0.05). See Table 2 for mean *d’* scores across SOA, Time and Group. Performance was better than expected at SOA 110 ms (we anticipated lower sensitivity at all shorter SOAs relative to longer SOAs), which is likely due to the poor ability to discriminate the beeps at 110 ms leading to reduced illusions. There was a significant interaction effect of Time × Group [*F*(1, 41) = 6.38, *p* = 0.015, $\eta_{\text{p}}^{2}$ = 0.14]. Simple effects analysis (using paired samples *t*-tests) was used to follow-up the interaction effect. *T*-tests revealed neither the older [*t*(22) = 1.99, *p* = 0.058, *d* = 0.41] nor younger group [*t*(19) = −1.95, *p* = 0.07, *d* = 0.44] significantly changed in sensitivity from pre to post-training. The significant interaction effect was driven by the trends for reduced sensitivity post-training for the older group and improved sensitivity post-training for the younger group. Both trends approached significance in the *t*-tests, with medium effect sizes. See Figure 3 for a scatterplot of individual *d’* scores.

There was also a significant interaction of SOA × Group [*F*(3, 123) = 10.63, *p* < 0.001, $\eta_{\text{p}}^{2}$ = 0.21]. The interaction of Time × SOA was not significant [*F*(3, 123) = 0.74, *p* = 0.53, $\eta_{\text{p}}^{2}$ = 0.02] nor was the three-way interaction [*F*(3, 123) = 1.65, *p* = 0.18, $\eta_{\text{p}}^{2}$ = 0.04]. Considering the skew in variance, an additional 2 (Group) × 2 (Time) non-parametric ANOVA was conducted on *d’* scores. The main effect of Group maintained statistical significance [*F*(1, 24.96) = 78.73, *p* < 0.001]. There was no significant main effect of Time [*F*(1, 24.51) = 0.11, *p* = 0.74] nor an interaction effect of Time × Group [*F*(1, 24.96) = 3.19, *p* = 0.09].

## Discussion

In this study, we aimed to utilize a paradigm that has been consistently successful with younger adults, simultaneity judgment (particularly the 2-IFC), to refine older adults’ multisensory perceptual abilities. We also tested participants’ performance in the SIFI pre- and post-training to assess the generalizability of any training effects. Three days of feedback training resulted in improved accuracy on the 2-IFC task in older adults, with pre-training accuracy on average slightly better than chance (56%) and post-training accuracy of 62%. As expected, the young adult group also significantly improved from a mean accuracy of 73% at pre-training to 76% post-training. Furthermore, both groups experienced a significant narrowing of the TWI, indicating computer-based feedback training is effective for improving audio-visual discrimination abilities in both older and young adults. This is in line with previous studies which found similar effects in young adults (Theves et al., 2020). They found that this improvement is associated with increased beta-band oscillations post-training, compared to pre-training.

Of interest is whether this training effect generalizes beyond the 2-IFC task itself. For both younger and older adults, we did not find a generalization of training to the SIFI; participants did not significantly improve (i.e. reduce) their susceptibility to the illusion post-training. However, it is worth noting both groups showed trends approaching significance, such that the older group had a trend of reduced sensitivity post-training and the young group showed a trend of improved sensitivity post-training. The mean trend for young adults is in line with previous experimental research on this age group, where SIFI performance improved post perceptual training (Powers et al., 2016).

The observed trend for older adults showing decreased sensitivity after training is unexpected, considering their improved performance on the 2-IFC task. Fatigue may explain this finding, as the SIFI task was always administered last during post-training measurements, which occurred on the third day of a 3-day consecutive testing protocol. Attention is considered an important factor in multisensory integration (Talsma et al., 2010; Talsma, 2015) and attention has been found to modulate performance on the SIFI (Mishra et al., 2010). It is plausible that fatigue may have impacted older adults’ attention during task performance, accounting for the trend for poorer performance post-training.

We argue the lack of generalization of training benefits for older adults could also be due to task difficulty of the 2-IFC task. While a significant narrowing of the TWI was found for older adults, many of the older group had the maximum window length of 300 ms (*n* = 19 at pre-training; *n* = 14 at post-training). This may indicate the SOA range was too short to capture a true reflection of TWI as several participants windows may have exceeded the 300 ms threshold. For example, Gieseler et al. (2018) in their study on older adults with mild hearing impairment estimated the average TWI at 70–220 ms for those who are not hearing-aid users and found enlarged windows for those who used hearing aids (TWI of 70–370 ms). While our study did not recruit individuals with mild hearing impairment and none of our participants were hearing aid users, it’s possible older adults show considerable variability in their TWI and a greater range of SOAs is needed to capture this wide spread. The proportion of correct responses in the 2-IFC, even at longer SOAs such as 250 and 300 ms, was approximately 60–70%, indicating participants found the task challenging, although not impossible to perform. The average TWI reported with TOJ (Setti et al., 2014) was lower than 200 ms, suggesting a difference in task difficulty. Further methodological reflections should be highlighted, particularly when comparing the 2-IFC with the TOJ staircase utilized in previous studies (Setti et al., 2014). While SJs show narrower TWI in older adults compared to TOJ performance, with no age difference (for sound-lagging stimuli; Chan et al., 2017); the 2-IFC, specifically, requires one to maintain in working memory two-pairs of stimuli and decide which one of the pairs was synchronous (or asynchronous). Considering that working memory capacity decreases with age (Wingfield et al., 1988), this task may produce a cognitive load that is difficult for older adults, as our older participants had close to chance accuracy (56%) at baseline. The effect of training may be undermined by task difficulty; although some level of challenge is necessary for perceptual learning (DeLoss et al., 2013).

It is possible that a more tailored approach is needed, whereby the individual thresholds are established before starting the training and the SOAs are modified accordingly (see discussion on adapting stimuli to participant’s sensory function in Hirst et al., 2020); alternatively a paradigm utilizing only one pair of stimuli (TOJ or SJ), i.e. potentially requiring lower working memory load could be more effective. Alternatively, it is possible that older adults, who rely more than young adults on previous knowledge in various tasks (Kathleen Pichora-Fuller, 2008; Maguinness et al., 2011; Chan et al., 2017), may show more limited improvements due to a decreased ability (or need) to update their implicit expectations, e.g. perceptual templates in such tasks. However, we propose that this is unlikely, considering that older adults have shown unisensory perceptual training abilities (e.g., tactile thresholds; Kalisch et al., 2008), and show a modulation of the SIFI, within participants, based on the number of SOAs, i.e., the information provided by the task. In addition, in the present study, a more comprehensive set of stimuli, including visual and auditory lead, could have offered more information to the individuals and, potentially, better training (see Chan et al., 2018). Finally, to account for possible lapses in sustained attention during training, a version of the SJ training like that of Yarrow et al. (2016) which offers the option of canceling trials due to inattention could be used in future studies. Canceled trials are repeated at the end of a trial block, therefore providing the possibility to participants of being potentially more confident about their judgments than in the present task. Further research is needed comparing such paradigms and the perceptual thresholds derived from them.

In their susceptibility to the SIFI, the present sample appears comparable to previous studies (Setti et al., 2014) with an average *d’* of 0.86 and 1, respectively. We found no effect of training in the susceptibility to the SIFI in young or older adults, but we note the young adults showed a trend for improved mean sensitivity post-training, a finding which approached statistical significance. The lack of an effect in older adults corresponds with previous research in the field, whereby perceptual training does not generalize to improvements in susceptibility to other perceptual illusions. The SJ (2-AFC) was not found to be predictive of susceptibility to the McGurk illusion in older adults in a lifespan study (de Boer-Schellekens and Vroomen, 2014; Stevenson et al., 2018). Similarly, SJ was not associated with susceptibility to the Stream Bounce illusion in Bedard and Barnett-Cowan’s study in older adults, while it was in younger adults (Bedard and Barnett-Cowan, 2016). Therefore it is possible that SJ training (2-AFC) generalizes to SIFI only in young adults, in line with the correlation found by Bedard and Barnett-Cowan (2016) between Stream Bounce and SJ in younger but not in older adults (but see Powers et al., 2016 for contrasting results). One result not in line with current literature is the better performance for older adults on SOA 70 ms compared to longer SOAs (i.e., they experience fewer illusions at SOA 70 ms). While this result counters our predictions as performance generally improves with longer SOAs, it can be explained by the poor auditory temporal resolution at SOA 70 ms in beep only conditions. It is plausible this reduced ability to detect auditory stimuli at SOA 70 ms could lead to reduced or non-experience of illusions. This result is also in line with recent findings on a sample of 3,955 individuals aged 50+ from The Irish Longitudinal Study on Aging (TILDA; Hernández et al., 2019). In this epidemiological study, participants were tested with a version of the SIFI comprising three SOAs; 70, 150, and 230 ms. Performance was significantly worse at 150 and 230 ms than 70 ms. However, while poorer auditory accuracy with an SOA of 70 ms can explain the lower susceptibility to the SIFI with the same SOA, the majority of participants are able to perceive the beeps and furthermore Gieseler et al. (2018) find older adults with mild hearing impairment show comparable susceptibility to the SIFI as normal hearing older adults of previous studies. Interestingly, amongst their sample of older adults with mild hearing impairment, they find greater susceptibility to the SIFI in hearing aid users compared to non-users, pointing to a complex association between hearing acuity and multisensory integration in aging (Gieseler et al., 2018). In light of this, the necessity of objective measures of participants’ hearing acuity in future perceptual training studies with older adults is acknowledged.

While we could not register an effect of training on the SIFI, it is worth mentioning that, older participants’ performance did not differ from the pre- to the post-training, supporting the claim that the SIFI is a useful tool to assess multisensory performance, robust to test/re-test for older participants.

As a limitation, we acknowledge that both fall-prone and non-fall-prone participants were included in our group of older participants, which may have led to additional variability in the overall findings, considering that fallers may have an extended audio-visual TWI (Setti et al., 2011; Stapleton et al., 2014). Further research is needed to discern whether perceptual training could reduce fallers’ TWI and potentially reduce falls-risk. Another limitation of this study is the use of visual leading stimuli only. This choice is justified by Powers et al. (2009) finding that the TWI was more malleable with visual leading stimuli, likely because it was larger in the first place.

Considering that multisensory integration is emerging as a novel factor in determining cognitive and functional aging (Setti et al., 2014; Chan et al., 2018; Murray et al., 2018), it is of paramount importance to find ways to improve multisensory processing efficiency, when it is compromised by the aging process. In the present study we focused on temporal discrimination abilities. Perceptual training is the traditional way to improve temporal discrimination, we adopted a paradigm which was effective for narrowing the TWI in young adults to determine whether it is suitable for older adults. Although this is a pilot study, not a randomized controlled trial, we argue that the task-specific benefits of 2-IFC training for healthy older adults found here, should promote further research into the best ways to train temporal abilities across the senses in older adults, taking into account cognitive load. Furthermore, we note the large effect size found, with a *post-hoc* power of 99% (effect size *f* = 0.55) of the effect of training on 2-IFC performance. Future studies should also investigate whether working memory capacity is associated with 2-IFC performance in older adults, as we suggest here. At present, there is limited evidence of generalized perceptual improvement through training in older adults. It should be noted however that the present study presents SOAs up to 300 ms and visual-lead only stimuli. Although in line with previous literature, it is possible that exposing and providing feedback to participants on a wider range of SOAs could have led to improvements beyond the task itself (i.e. on SIFI performance). Whether a staircase procedure is necessary, although often more daunting on participants time and effort, also remains an open question.

The limitations of a small sample should be acknowledged; therefore, we cannot exclude the possibility that 2-IFC may lead to significant benefits for the SIFI or other multisensory tasks in a larger sample. However, our sample is in line with the sample size of published studies on young and older adults’ perceptual learning. Importantly, we provide simultaneous data for young and older adults, allowing comparisons to be made in the outcomes of perceptual training for these two groups. Our research extends previous work on audio-visual perceptual training in younger populations (Powers et al., 2009, 2016; Theves et al., 2020) to older adults, providing evidence that 3 days of 2-IFC training can improve older adults’ task performance and reduce their TWI, but this training does not generalize beyond the 2-IFC task (at least not to the SIFI task). We argue that the task-specific training results observed in this study are useful to inform future research to maximize benefits for participants in multisensory perceptual learning tasks, as ethical considerations should be taken into account before undertaking large trials with older adults, utilizing paradigms that have limited success in pilot work. This research contributes to mounting evidence that multisensory processing deficits in older adults’ can be modified through targeted perceptual training. Perceptual training could potentially represent a novel intervention avenue for populations whose impairments are associated with underlying multisensory processing difficulties (e.g. fall-prone older adults; Mahoney et al., 2019). Whether training produces benefits for functional abilities underpinned by multisensory processing (i.e. balance control) remains to be explored.

## Data Availability Statement

The raw data supporting the conclusions of this article will be made available by the authors, without undue reservation, to any qualified researcher.

## Ethics Statement

The studies involving human participants were reviewed and approved by the School of Applied Psychology Ethics Committee University College Cork. The patients/participants provided their written informed consent to participate in this study.

## Author Contributions

AS and JC conceived and designed the study. JO’B completed data collection. All authors contributed to data analysis and the writing and editing of this manuscript.

## Conflict of Interest

The authors declare that the research was conducted in the absence of any commercial or financial relationships that could be construed as a potential conflict of interest.

## Supplementary Material

Supplementary Figure 1 Graph representing calculation of Temporal Window of Integration (TWI) for one participant. Participant data on 2-IFC task at pre-training is fitted with a 3rd order polynomial probability curve to calculate estimated window size. 126.10 ms is width of window for participant below. Black lines mark the point halfway between the participant’s lowest accuracy score and 100% (i.e. 75%).

Supplementary Figure 2 Mean proportion of correct responses per SOA in each day of training, for the older (1a) and younger (1b) groups.

Supplementary Table 1 Group means on unisensory conditions of the SIFI at pre-training (standard deviation in parentheses).

Supplementary Data Sheet 1 Data and results for asynchronous training group.
