# Supplementary figures and images for: Audio-Visual Training in Older Adults: 2-Interval-Forced Choice Task Improves Performance
Source: Front Neurosci. 2020 Nov 12;14:569212. doi: 10.3389/fnins.2020.569212 (PMC7693639; doi:10.3389/fnins.2020.569212)

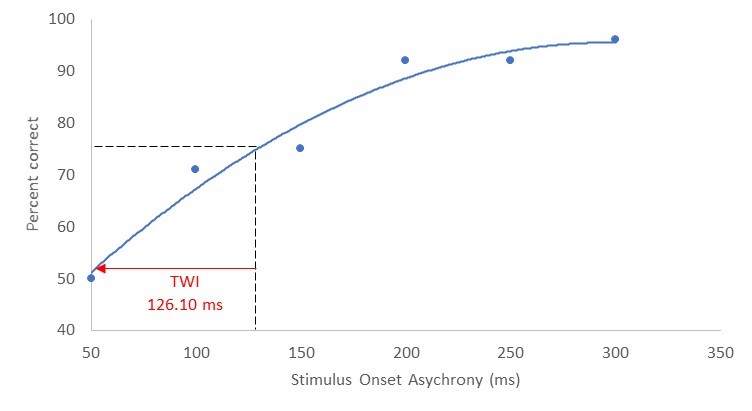

Supplement: Supplementary Figure 1 — Graph representing calculation of Temporal Window of Integration (TWI) for one participant. Participant data on 2-IFC task at pre-training is fitted with a 3rd order polynomial probability curve to calculate estimated window size. 126.10 ms is width of window for participant below. Black lines mark the point halfway between the participant’s lowest accuracy score and 100% (i.e. 75%). [file Image_1.jpg]
