# Supplementary material for: Audio-Visual Training in Older Adults: 2-Interval-Forced Choice Task Improves Performance
Source: Front Neurosci. 2020 Nov 12;14:569212. doi: 10.3389/fnins.2020.569212 (PMC7693639; doi:10.3389/fnins.2020.569212)
Supplement: Supplementary Figure 2 — Mean proportion of correct responses per SOA in each day of training, for the older (1a) and younger (1b) groups. [file Image_2.pdf]

Figure S2. Mean proportion of correct responses per SOA in each day of 2-IFC training, for the older (**A**) and younger (**B**) groups.

A)

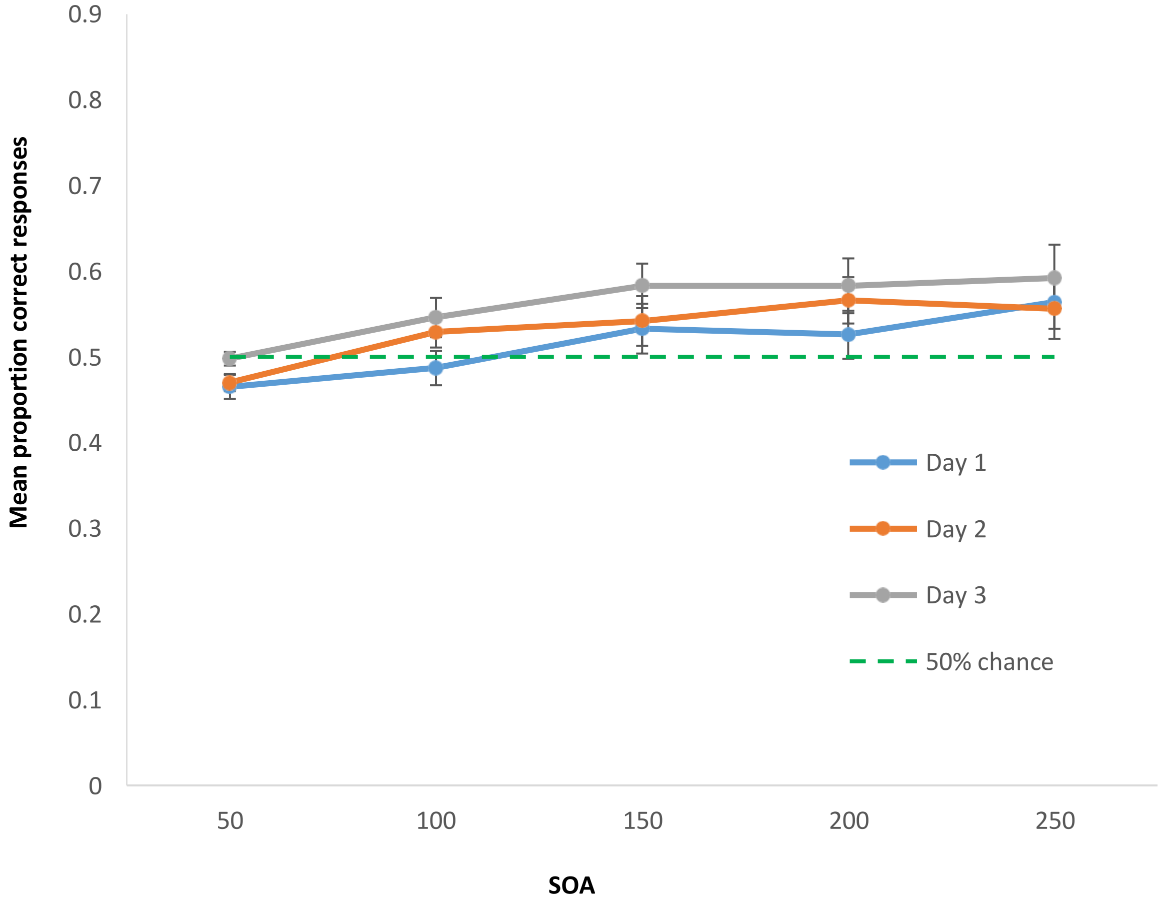

B)

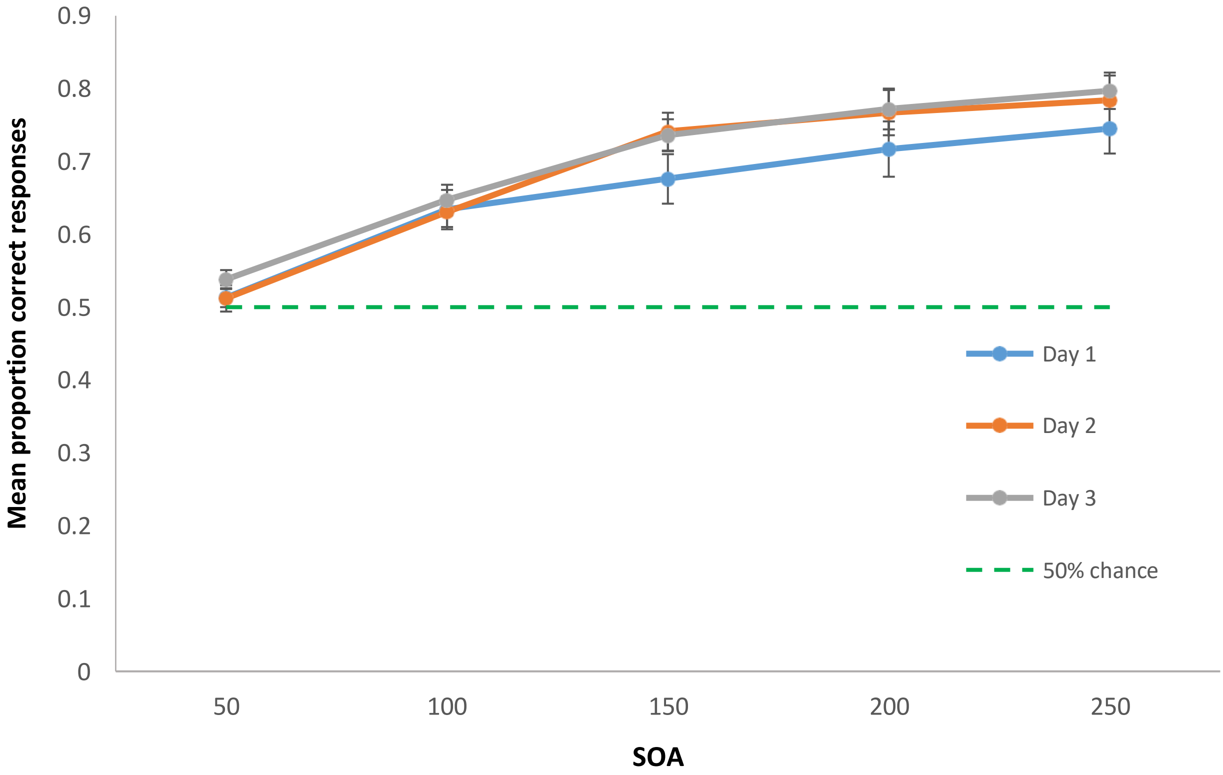

0.9
0.8
0.7
0.6
0.5
0.4
0.3
0.2
0.1
0
Mean proportion correct responses
50
100
150
200
250
SOA
Day 1
Day 2
Day 3
50% chance
