# Supplementary material for: Audio-Visual Training in Older Adults: 2-Interval-Forced Choice Task Improves Performance
Source: Front Neurosci. 2020 Nov 12;14:569212. doi: 10.3389/fnins.2020.569212 (PMC7693639; doi:10.3389/fnins.2020.569212)
Supplement: Supplementary Table 1 — Group means on unisensory conditions of the SIFI at pre-training (standard deviation in parentheses). [file Table_1.docx]

| Table S1 |  |  |  |
| --- | --- | --- | --- |
| *Descriptive characteristics per older training group* | | | |
|  | Synchronous | Asynchronous | p value |
| *N* | 23 | 20 |  |
| Age | 74.17 (6.28) | 70.56 (6.07) | 0.07 |
| Gender | M: 13 (56.52%),  F: 10 (43.48%) | M: 3 (15%),  F: 15 (85%) | 0.01* |
| SMMSE | 28.67 (1.46) | 28.4 (1.96) | 0.69 |
| IPAQ | 4632.67(4331.63) | 2996.69  (2405.31) | 0.18 |
| Fallers (*n*) | 11 (42.85%) | 12 (51.85%) | 0.55 |
