# Supplementary material for: Audio-Visual Training in Older Adults: 2-Interval-Forced Choice Task Improves Performance
Source: Front Neurosci. 2020 Nov 12;14:569212. doi: 10.3389/fnins.2020.569212 (PMC7693639; doi:10.3389/fnins.2020.569212)
Supplement: Supplementary Data Sheet 1 — Data and results for asynchronous training group. [file Data_Sheet_1.docx]

**Data Sheet S1.** Data and results for asynchronous training group

A sample of 20 community-dwelling older adults (*M* = 70.56, *SD* = 6.07) were tested on a variation of the perceptual training used in the current study. This additional group were labelled the Asynchronous group. They completed the same pre- and post-training measures as our older and young adult groups but their training protocol was slightly different. Instead of being asked to identify the simultaneity of stimuli, participants were asked to identify which stimulus pair were presented asynchronously. They also received feedback during this training.

# Rationale

It is important to note that there are potentially substantial differences between the paradigms successfully utilised with young adults (Powers et al., 2009; 2012), and the partially successful one with older adults (Setti et al. 2014). The former is based on a simultaneity judgement (SJ), while the latter is based on a temporal order judgement. In two studies (Basharat et al., 2018; Bedard and Barnett-Cowan, 2016), the audio-visual SJ was found to be easier for older adults than the TOJ task, as indicated by a narrower TWI in the SJ task than in the TOJ task. In addition, neither the TOJ or SJ were related to the susceptibility to the stream-bounce illusion (Bedard and Barnett-Cowan, 2016). Simultaneity and temporal order may in fact be implemented by different brain networks (Basharat et al., 2018), different processes, and response biases are thought to underlie the two types of tasks (Spence and Parise, 2010). The crucial differences can reside in participants’ attention or their decision-making process in relation to task demands (García-Pérez and Alcalá- Quintana, 2012). The question arises whether one could utilise the same SJ perceptual stimuli, and direct participants’ attention to the temporal discrepancy, instead of the synchrony, with differential training effects.

# Method

The pre-training and post-training measures were identical to that of the main experiment. However, the instructions during training differed for this asynchronous group. Instead of focusing on the synchrony of stimuli, participants were tasked with identifying the stimulus pair in which the stimuli were presented asynchronously (i.e. were not presented simultaneously). Descriptive characteristics of this sample compared to our older adult experimental group (i.e. synchronous training) are presented in Table S1.
